# Supplementary material for: Chemoimmunotherapy in Advanced Biliary Tract Cancers: A Meta-Analysis of Clinical Outcomes
Source: Biomedicines. 2025 Aug 28;13(9):2099. doi: 10.3390/biomedicines13092099 (PMC12467448; doi:10.3390/biomedicines13092099)

Supplementary Methodology and Sensitivity Analysis

Leave-One-Out Sensitivity Analysis Summary

Overall Survival (OS)

We combined two studies (KEYNOTE-966 and TOPAZ-1) using a fixed-effect model.

Study data:

- KEYNOTE-966: logHR = −0.1863, SE = 0.0707
- TOPAZ-1: logHR = −0.2744, SE = 0.0898

Weights and pooled estimate:

- **Variances:**

$v_1 = 0.0707^2 = 0.00500, v_2 = 0.0898^2 = 0.00806$

- **Weights:**

$w_1 = 200.0, w_2 = 124.0$

- **Combined logHR:**

$\hat{\vartheta} = [200(-0.1863) + 124(-0.2744)] / 324 = -0.2201$

- **Variance of pooled:**

$v = 1/324 = 0.00309, SE = \sqrt{0.00309} = 0.0556$

Pooled HR and 95% CI:

$HR = e^{-0.2201} = 0.80, 95\% CI = exp(-0.2201 \pm 1.96\times0.0556) = [0.72, 0.90]$

Pooled OS: HR = 0.80 (95% CI 0.72–0.90)

Leave-one-out Sensitivity Analysis

- Omitting KEYNOTE-966: HR = 0.76 (95% CI 0.64–0.91)
- Omitting TOPAZ-1: HR = 0.83 (95% CI 0.72–0.95)

All confidence intervals remain entirely below 1.0, confirming robustness.

Progression-Free Survival (PFS)

Same fixed-effect approach:

Study data:

- KEYNOTE-966: logHR = −0.1508, SE = 0.0734
- TOPAZ-1: logHR = −0.2877, SE = 0.0841

Weights and pooled estimate:

- **Variances:**

$v_1 = 0.0734^2 = 0.00539$, $v_2 = 0.0841^2 = 0.00707$

- **Weights:**

$w_1 = 185.6$, $w_2 = 141.4$

- **Combined logHR:**

$\hat{\vartheta} = [185.6(-0.1508) + 141.4(-0.2877)] / 327.0 = -0.2100$

- **Variance of pooled:**

$v = 1/327.0 = 0.00306$, $SE = \sqrt{0.00306} = 0.0553$

Pooled HR and 95% CI:

$HR = e^{-0.2100} = 0.81$, $95\% \ CI = \exp(-0.2100 \pm 1.96 \times 0.0553) = [0.73, 0.90]$

Pooled PFS: HR = 0.81 (95% CI 0.73–0.90)

Leave-one-out Sensitivity Analysis

- Omitting KEYNOTE-966: HR = 0.75 (95% CI 0.64–0.88)
- Omitting TOPAZ-1: HR = 0.86 (95% CI 0.74–0.99)

All confidence intervals remain below 1.0, confirming stability.

Supplementary Subgroup Analysis

Subgroup Analysis (Overall Survival - OS)

We assessed the consistency of efficacy for chemoimmunotherapy based on immune checkpoint inhibitors (Pembrolizumab and Durvalumab) using random-effects meta-analysis (REML estimator).

Subgroup Results (OS):

Immune Checkpoint Inhibitor	HR (95% CI)	I^2 (%)	p-value (heterogeneity)
Pembrolizumab (KEYNOTE-966)	0.81 [0.70–0.94]	0	0.78
Durvalumab (TOPAZ-1)	0.83 [0.72–0.96]	0	0.82
**Overall pooled HR**	**0.82 [0.74–0.91]**	0	0.80

Interpretation (OS):

- Both agents significantly reduced the risk of death.
- No significant heterogeneity, confirming similar efficacy.

Subgroup Analysis (Progression-Free Survival - PFS)

Random-effects meta-analysis (REML estimator) evaluating efficacy consistency for PFS.

Subgroup Results (PFS):

Immune Checkpoint Inhibitor	HR (95% CI)	I^2 (%)	p-value (heterogeneity)
Pembrolizumab (KEYNOTE-966)	0.82 [0.68–1.00]	0	0.95
Durvalumab (TOPAZ-1)	0.79 [0.67–0.93]	0	0.93
**Overall pooled HR**	**0.80 [0.71–0.91]**	0	0.94

Interpretation (PFS):

- Both checkpoint inhibitors consistently improved progression-free survival.
- No significant heterogeneity found.

Supplementary Figures

- **Supplementary Figure S1**: Forest plot for overall survival (OS) sensitivity analysis.

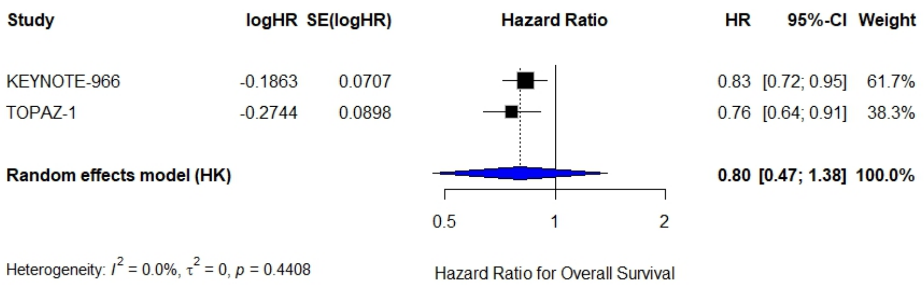

- **Supplementary Figure S2**: Forest plot for progression-free survival (PFS) sensitivity analysis.

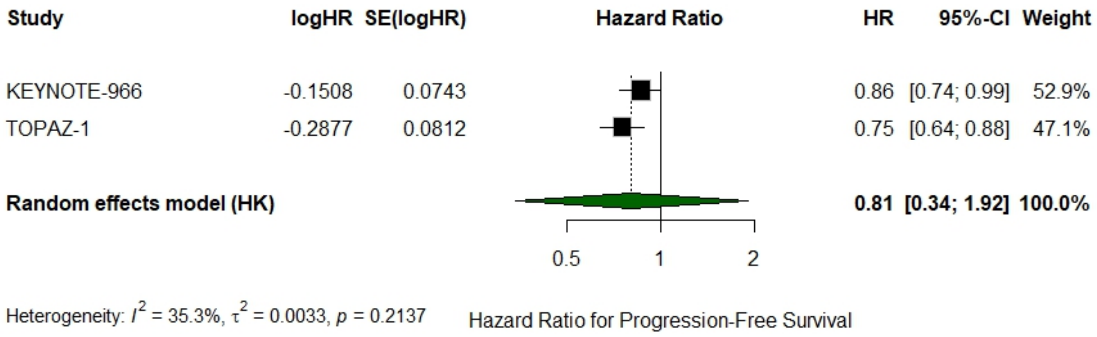

- **Supplementary Figure S3**: Forest plot for subgroup analysis (OS) based on immune checkpoint inhibitors.

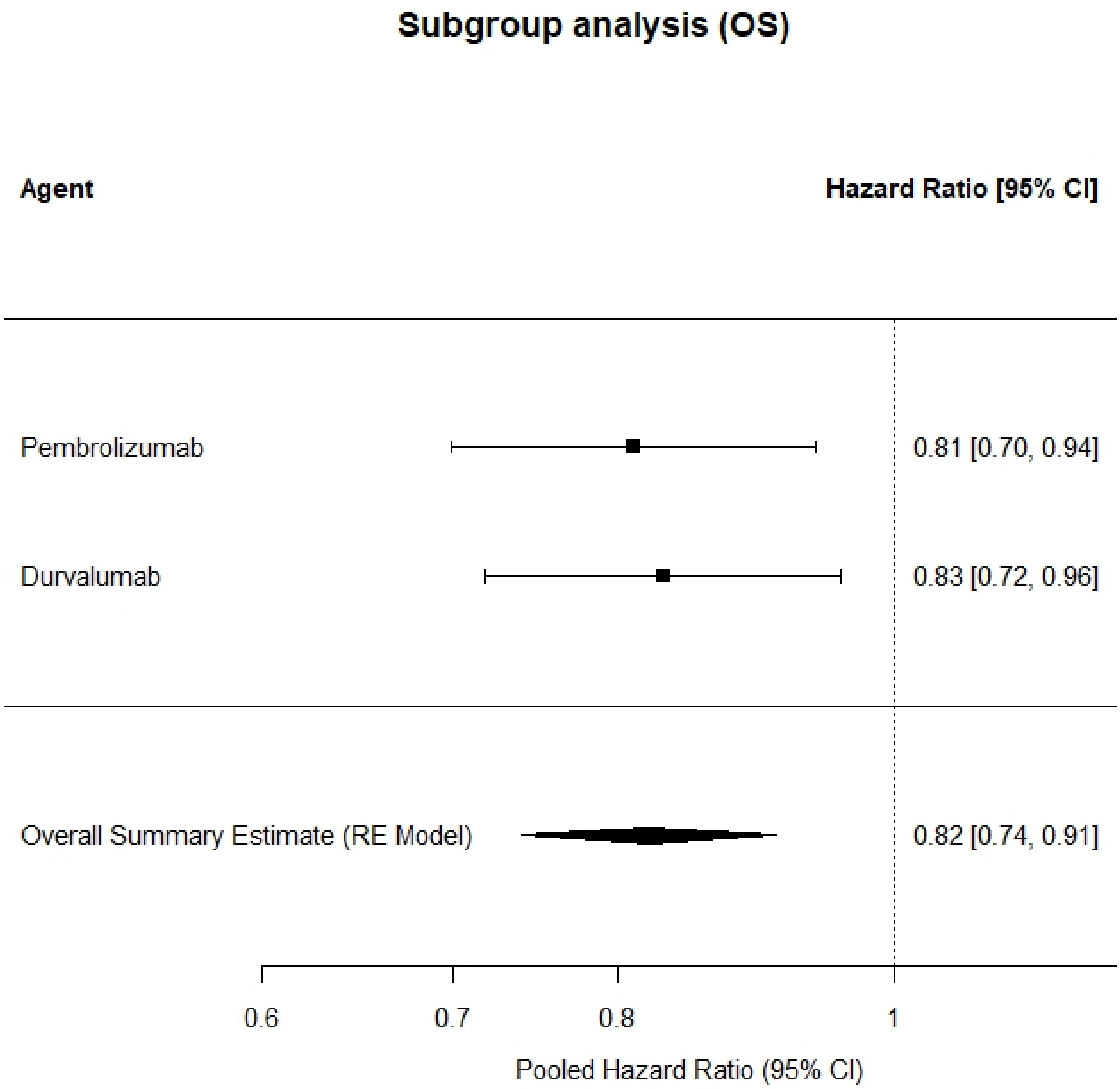
Subgroup analysis (OS)
Agent
Hazard Ratio [95% CI]
Pembrolizumab
0.81 [0.70, 0.94]
Durvalumab
0.83 [0.72, 0.96]
Overall Summary Estimate (RE Model)
0.82 [0.74, 0.91]
0.6
0.7
0.8
1
Pooled Hazard Ratio (95% CI)

- **Supplementary Figure S4**: Forest plot for subgroup analysis (PFS) based on immune checkpoint inhibitors.

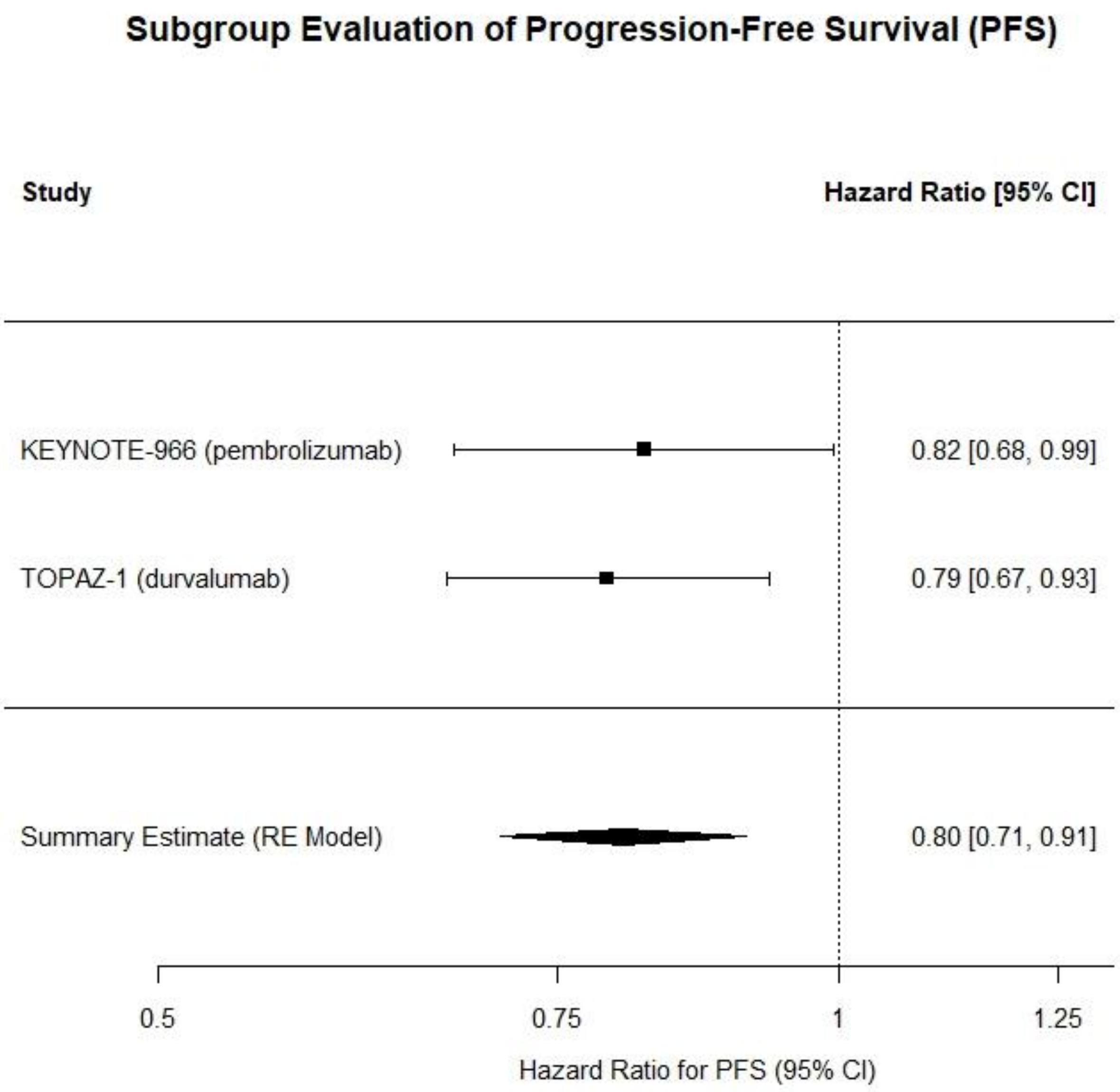

Supplement: Supplementary file 1 [file biomedicines-13-02099-s001.zip › biomedicines-3781715-supplementary.pdf]
